# Emerging biomarkers in metastatic urothelial carcinoma: tumour mutational burden, PD-L1 expression and APOBEC polypeptide-like signature in a patient with complete response to anti-programmed cell death protein-1 inhibitor

**DOI:** 10.3332/ecancer.2021.1306

**Published:** 2021-10-22

**Authors:** Marcello Moro Queiroz, Zenaide Silva de Souza, Aline Bobato Lara Gongora, Felipe de Galiza Barbosa, Carlos Alberto Buchpiguel, Marilia Germanos de Castro, Mariana Petaccia de Macedo, Rafael Ferreira Coelho, Ethan Samuel Sokol, Anamaria Aranha Camargo, Diogo Assed Bastos

**Affiliations:** 1 Oncology Center, Hospital Sírio-Libanês (HSL), Rua Dona Adma Jafet, 91, São Paulo, SP, 01308-050, Brazil; 2 Department of Diagnostic Imaging and Nuclear Medicine, Hospital Sírio-Libanês (HSL), Rua Dona Adma Jafet, 91, São Paulo, SP, 01308-050, Brazil; 3 Department of Pathology, Hospital Sírio-Libanês (HSL), Rua Dona Adma Jafet, 91, São Paulo, SP, 01308-050, Brazil; 4 Instituto do Câncer do Estado de São Paulo, Av Dr Arnaldo, 251 - Cerqueira César, São Paulo, SP, 01246-000, Brazil; 5 Cancer Genomics Research, Foundation Medicine Inc., 150 Second St, Cambridge, MA 02141, USA; a https://orcid.org/0000-0001-5789-3397; b https://orcid.org/0000-0002-9743-191X; c https://orcid.org/0000-0002-2167-8166; d https://orcid.org/0000-0002-3986-1778; e https://orcid.org/0000-0003-0956-2790; f https://orcid.org/0000-0001-8882-4650; g https://orcid.org/0000-0002-0434-7605; h https://orcid.org/0000-0002-2988-0537; i https://orcid.org/0000-0002-6076-9597; j https://orcid.org/0000-0003-2480-353X

**Keywords:** bladder cancer, metastatic, immunotherapy, biomarkers, tumour mutational burden, PD-L1

## Abstract

Immunotherapy has recently been incorporated into the treatment guidelines for metastatic urothelial carcinoma. Nevertheless, the role of prognostic and predictive biomarkers in this setting is not completely defined. To date, PD-L1 expression and a high tumour mutational burden (TMB) seem to predict better responses to immune checkpoint inhibitors, but patients without these biomarkers may still respond to immunotherapy. There are some caveats regarding these biomarkers, such as lack of standardisation of techniques, tumour heterogeneity and other factors influencing the tumour microenvironment. Genomic signatures are other promising emerging strategies. We hereby discuss the management of a 70-year-old man with a metastatic recurrence of urothelial carcinoma within 1 year after neoadjuvant chemotherapy and radical cystectomy. Tumour next-generation sequencing showed a high TMB and a *CD274* (PD-L1) amplification. The patient was treated with pembrolizumab and achieved a complete response.

## Introduction

Bladder cancer is the fourth most common type of cancer in American men, with an estimate of 83.730 new cases and 34.130 deaths in the United States in 2021 [1]. Worldwide, there have been 549,000 new cases and 200,000 deaths due to bladder cancer in 2018 [2]. Cigarette smoking, occupational exposure to chlorine or arsenic and infectious agents are well established risk factors for this type of cancer. The exposure to certain drugs, such as cyclophosphamide and phenacetin-containing analgesics in high doses is related to the development of bladder cancer in humans [3].

Urothelial carcinoma is by far the most common histology of bladder cancer [4]. Muscle-invasive urothelial carcinoma is an aggressive disease, with a 5-year cancer-specific mortality of 37%–67% [5]. In metastatic bladder cancer, the scenario is even more discouraging: despite the high first-line response rates with platinum-based chemotherapy, the median overall survival (OS) varies between 11 and 16 months with conventional chemotherapy [6–9].

Over the last few years, cancer treatment is changing drastically with the development of new targeted therapies and immunotherapy. This paradigm-shift in cancer treatment includes urothelial cancer. As of September 2021, four immune checkpoint inhibitors (ICIs) involving programmed cell death protein-1 (PD-1)/programmed death ligand-1 (PD-L1) pathway have been approved by Food and Drug Administration (FDA) for second-line treatment of metastatic urothelial cancer (mUC) [10]. More recently, avelumab, an anti-PD-L1, was approved for maintenance treatment for patients with no disease progression after first-line chemotherapy [11, 12] and Pembrolizumab/Atezolizumab were granted approval for first-line cisplatin ineligible patients [13]. Although these advances represent encouraging results, many efforts have been devoted towards discovering biomarkers that can be used in clinical practice as predictive markers of treatment response. Some of the molecular determinants that have been studied in other types of cancer and are currently being assessed in bladder cancer are PD-L1 expression, DNA mismatch-repair deficient (dMMR), DNA damage repair gene alterations [14], tumour mutational burden (TMB) and tumour-infiltrating lymphocytes (TILs) [15].

Here we reviewed the newest data about biomarkers of response to ICI and described a case report of a patient with metastatic bladder cancer whose tumour next-generation sequencing (NGS) showed a high TMB and a *CD274* (PD-L1) amplification, and who presented a complete response (CR) after treatment with an ICI, in order to illustrate this subject.

## Case report

A 70-year-old man presented haematuria and a cystoscopy for investigation demonstrated a muscle-invasive urothelial carcinoma. A staging positron emission tomography/computed tomography with 2-deoxy-2[18-F]-fluoro-D-glucose (18-F FDG PET/CT) revealed an increased uptake on a parietal thickening of the right bladder wall with densification of the perivesical adipose tissue (standardised uptake values maximum (SUVmax): 12.9) and on right external iliac enlarged lymph nodes (LN), up to 3.0 cm (SUVmax: 20.1).

**Figure 1 (coloured).**
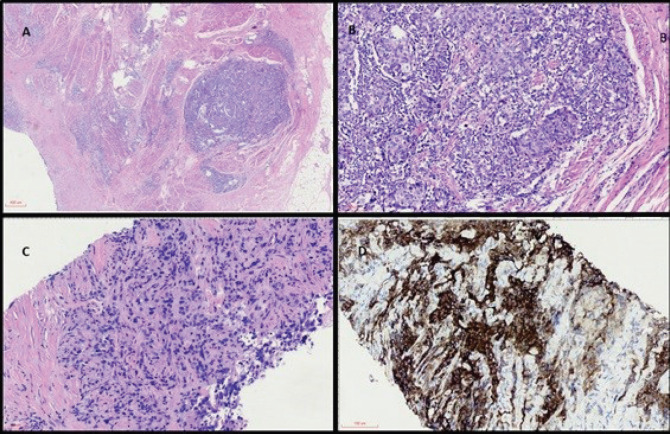
(a): Panoramic view of haematoxylin and eosin (H&E) stained sections of cystectomy specimen showing a highly infiltrative neoplasm through the vesical muscular wall (40×). (b): H&E high power view analysis of cystectomy (200×) with invasive carcinoma in muscular propria and intratumoral lymphocyte infiltration. (c): H&E analysis of metastatic lymph node with carcinoma (200×). (d): IHC with strong and diffuse positivity for PD-L1 22C3.

He then started on cisplatin-based chemotherapy with dense-dose methotrexate, vinblastine, doxorubicin and cisplatin (MVAC) for six cycles, presenting a partial response on imaging. After the last cycle, a radical cystoprostatectomy was performed and pathology showed a poor response to neoadjuvant chemotherapy (ypT3N2) (Figure 1). He was subsequently managed with close clinical and image follow-up.

Ten months after the cystoprostatectomy, an 18-F FDG PET/CT showed an increased uptake in mesorectal and inferior mesenteric LN (SUVmax: 7.9) and also in a nodule located on the right ischiatic foramen (SUVmax: 10.4). A biopsy of one of the suspicious LN confirmed metastatic urothelial carcinoma. The PD-L1 22C3 pharmDx immunohistochemistry (IHC) showed a Combined Positive Score (CPS) of 100. The paraffin-embedded tumour blocks from the LN metastasis were sent to FoundationOne CDX test. A NGS comprehensive panel was performed and showed a TMB of 18 muts/Mb, along with *CD 274* (PD-L1) amplification, *ERBB2* amplification, *PTCH1* E1183*, *CDKN1A* R48*, *JAK2* amplification*, KDM6A* V1113FS*7, *MUTYH* splice site 347-1G>C, *PAX5* rearrangement exon 2, *RB1* R787*, *TERT* promoter-146 C>T and *TP53* R282W. Microsatellite status was stable and there were no *FGFR* alterations. An analysis of tumour mutation trinucleotide context revealed a dominant apolipoprotein B mRNA editing catalytic polypeptide-like (APOBEC) signature [16].

Due to the short time between exposure to MVAC chemotherapy and progression of disease, the poor previous response to platinum-based therapy and NGS results with biomarkers indicating a higher possibility of response to immunotherapy (high TMB and high PD-L1 expression), the patient was started on treatment with pembrolizumab.

**Figure 2.**
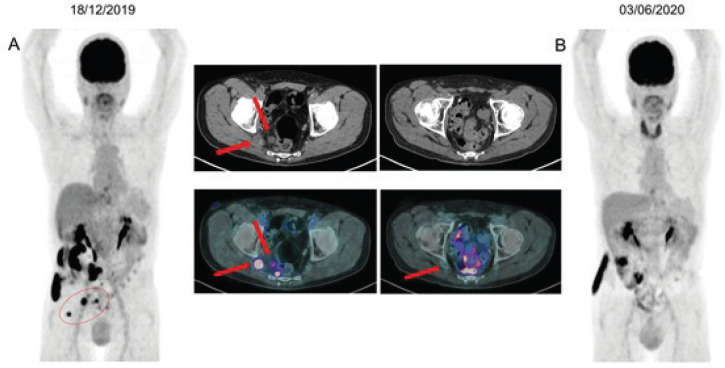
(a): 18F-FDG PET/CT imaging baseline before the initial therapy with pembrolizumab, showing mesorectal and inferior mesenteric LN, measuring up to 1.7 cm (SUVmax: 7.9). Nucleus centred on the right sciatic foramen measuring 2.0 cm (SUVmax: 10.4), maintaining close contact with the sciatic roots on this side. (b): Restaging after seventh cycle demonstrating resolution of glycolytic hypermetabolism in those previous LN.

An 18-F FDG PET/CT performed after the third cycle showed resolution of the glycolytic hypermetabolism in the mesorectal and inferior mesenteric LN and reduction in the right ischial foramen nodule. A new image after the seventh cycle demonstrated a CR (Figure 2), which was sustained after 9 months of treatment. The plan is maintenance of pembrolizumab until any limiting toxicity or disease progression. To the date of this publication, the patient remains with an ongoing CR with pembrolizumab.

### Biomarkers of immunotherapy response in metastatic urothelial carcinoma

Bladder cancer has a well-recognised course. Even when only non-muscle invasive bladder cancer is shown on the initial transurethral resection of bladder tumour, 50%–70% will develop multiple recurrences and 10%–20% will progress to muscle-invasive bladder cancer, conferring a worse prognosis [17].

Over the last 30 years, treatment regimens for mUC have remained almost unchanged [18, 19], favouring the use of platinum-based chemotherapies as first-line treatment, with no clear preferred option for second line [7]. However, with the development of PD-1/PD-L1 ICHs and targeted therapies, this paradigm has changed over the past few years [20].

In 2016, Atezolizumab was approved in second-line treatment of mUC by the FDA. This approval was based on the IMvigor 210 trial [10, 21]. Since then, four other anti-PD-1/PD-L1 therapies have been approved (Nivolumab, Durvalumab, Avelumab and Pembrolizumab) in the same scenario. Pembrolizumab is the only one with positive OS results in the second-line setting in a randomised phase III trial [22]. Most recently, atezolizumab and pembrolizumab were granted approval for first-line therapy in cisplatin-ineligible patients with overexpression of PD-L1. However, in June 2018, the FDA limited the use of both treatments in monotherapy for mUC based on a decreased survival when compared to platinum-based chemotherapy in patients who have not received prior therapy and have low expression of PD-L1. As a result, Pembrolizumab was indicated in cisplatin-ineligible patients and whose tumours express CPS ≥ 10 or chemotherapy-ineligible patients, regardless of PD-L1 expression. For Atezolizumab, the indication was for cisplatin-ineligible patients with tumour infiltrating immune cells (IC) ≥ 5% of the tumour area and chemotherapy-ineligible patients regardless of PD-L1 [13, 23, 24]. Moreover, avelumab has shown significant benefit as maintenance therapy after not progressing to induction with first-line platinum-based chemotherapy, with improvement in OS and manageable side effects, and was recently FDA approved in this setting [11, 12].

These new therapies have demonstrated significant anti-tumour activity, with a median survival varying from 7 to 10.3 months, and an overall response rate (ORR) between 15% and 23% in the second-line setting (compared with the 10% historical controls) [25]. In this same scenario, 5% and 7% of all patients achieved CR with Atezolizumab and Pembrolizumab, respectively, with a median time to response of 2.1 months with both ICI [22, 26].

After the publication of trials evaluating the response to ICI in many mUC scenarios, and their following approval by the FDA, cost-effectiveness analysis started to be made due to the high prices related to these new medications. For example, the cost-effectiveness of second-line Pembrolizumab was evaluated against chemotherapy or Atezolizumab in an American population, based on extrapolated results from KEYNOTE-045 and IMvigor-211. Results showed that despite the study limitations and at a $100.000 willingness-to-pay threshold scenario, pembrolizumab and atezolizumab had a 66% and 100% probability of being cost-effective against chemotherapy, respectively [27]. Pembrolizumab was also evaluated against carboplatin-based chemotherapy in a PD-L1 positive first-line scenario using data from KEYNOTE-052. In this study, the use of the anti-PD-1 ICI resulted in a mean gain of 2.58 life years, 2.01 quality-adjusted life-years and additional costs of $158.561, with a cost-effectiveness ratio of $78.925/quality-adjusted life-year, when compared to carboplatin plus gemcitabine [28]. These analyses demonstrate that the use of this medication may be feasible, especially for patients that are likely to respond compared to other options (biomarker-driven strategy). The cost-effectiveness of pembrolizumab in second-line scenario was also suggested in the Swedish population; however, another study indicated that this medication can be cost-effective in the United States, but not in other countries [29, 30].

Nevertheless, concern has been raised on how to predict which patients would have better responses to ICI, since most patients unfortunately do not respond to this approach. In the pursuit of an answer to this question, many studies are evaluating possible biomarkers of response, with a special attention to PD-L1 expression evaluation by IHC and TMB [31, 32].

## PD-L1 expression by IHC

PD-L1 expression evaluated by IHC in bladder cancer is associated with increased all-cause mortality and worse pathologic stage at resection, suggesting that high levels of PD-L1 expression may indicate more aggressive disease. The expression of this biomarker may be a prognostic factor and also predictive of response to PD-1/PD-L1 therapy [33, 34]. However, across clinical trials regarding mUC, PD-L1 assays and clinical results varied significantly, showing the challenge of using PD-L1 alone as a predictive biomarker. One explanation to this discrepancy is the lack of standardisation for PD-L1 detection between the studies. Of note, there are four distinct assays for PD-L1 IHC scoring used in the trials, with different scoring compartments for each specific therapy (some use tumour cell expression and others combine with immune cell expression) and distinct cut off values of PD-L1 expression (varying from 1%, 5%, 10%, and 25%). Moreover, PD-L1 as a tumour marker is dynamic over time and space, and a single biopsy may not be enough to represent the immune landscape in its entirety [25]. Most importantly, PD-L1 status is only a single factor in the tumour microenvironment. Other important features might more accurately segregate ‘hot’ from ‘cold’ tumours [35, 36].

## PD-L1 (CD274) amplification

*PD-L1 (CD274)* amplification is relatively common in certain lymphomas and is associated with a high susceptibility to PD-1 blockade, but data are limited regarding solid tumours [37, 38]. In a retrospective study from Goodman *et al* [38], the prevalence of PD-L1 amplification in 118,187 solid tumours samples from the Foundation Medicine database was 0.7%, and this alteration did not always correlate with high-positive PD-L1 expression by immunohistochemical analysis. In patients with PD-L1 amplified solid tumours that were treated with ICI, the objective response rate was 66.7% (6/9), with a median progression-free survival of 15 months [38]. However, prospective studies with larger number of patients are needed to confirm the correlation between PD-L1 amplification and responses to ICI, and there are no studies in urothelial cancer until now.

## Tumour mutational burden

Regarding the TMB analysis, a variety of clinical studies have shown that patients with higher TMB experienced better prognosis, longer survival and greater response rates following treatment with ICI compared to those who have lower TMB levels [15, 39]. In patients with non-muscle invasive urothelial carcinoma treated with Bacillus of Calmette–Guérin immunotherapy, a high TMB was significantly associated with a higher response rate and recurrence-free survival [40]. Studies from melanoma and non-small cell lung cancer (NSCLC) suggest that the mutational load may potentially predict response more robustly than PD-L1 IHC, presence of TILs or clinical variables [41, 42]. When evaluating gene expression profiles and immune cell infiltration signatures in bladder cancer, Wu *et al* [43] indicated that TMB is closely related with immune microenvironment, suggesting that higher TMB tends to promote the infiltration of T cells and natural killer (NK) cells into the tumour microenvironment and thus, patients may achieve a more favourable prognosis. Beyond that, Wang *et al* [15] analysed the gene-set from The Cancer Genome Atlas (TCGA) database and correlated the expression of cancer-testis antigens (CTAs) with higher TMB patients. CTAs are a group of immunogenic proteins that are aberrantly activated in a variety of cancer types and are important targets for developing cancer immunotherapy.

Nevertheless, like PD-L1 expression, TMB has its limitations as a predictive biomarker. Many factors influence TMB assessment, which includes preanalytical factors, choice of the assay and methods of reporting (the last one can change depending on the assays and centres where the tests are done) [44]. The definition of what is considered a TMB high lacks standardisation between studies, as the cut-off value may vary depending on the publication, and the methods for calculating are inconsistent depending on the methodology used (for example, including or not synonymous mutations and Indels in the equation). Another problem is the technical limitation, depending on which NGS panel is used, because some of them may not include analysis of gene fusions, truncations and translocations, limiting the treatment evaluation when these alterations are present. Also, TMB is not constant during the whole treatment, meaning that its value may vary depending on which drugs the patient has been exposed to, thus demonstrating that a single biopsy during the whole treatment may not be sufficient to predict response to subsequent therapies. Lastly, germline variants may interfere with the results, as the techniques may not correctly filter common germline single nucleotide polymorphisms [25, 45].

Defects in DNA repair mechanisms, like microsatellite instability (MSI) and *POLE* (DNA polymerase epsilon) mutations, may work as surrogate measures for TMB and have emerged as potential biomarkers in the literature [25, 46]. Patients in the metastatic scenario with MSI high and dMMR presented a better response rate when treated with pembrolizumab. Also, the phase II KEYNOTE 158 established the link between TMB-high status and improved ORR to anti-PD-1 in patients with various solid tumours. Based on these results and many others, the FDA granted Pembrolizumab agnostic approval for patients with solid tumours that carry the defects in dMMR, MSI high or TMB-high (≥ 10 mutations/megabase) in the second-line scenario [47–50].

### Other biomarkers

In an attempt to override the limitations in finding a reliable biomarker, there are numerous examples of studies across solid tumour types including head and neck squamous cell cancer, NSCLC, melanoma, and urothelial cancer exploring correlation between composite markers and response to anti-PD-1 [51, 52]. For example, in the subgroup analysis of the phase III IMvigor 130, a study in locally advanced or MUC, Atezolizumab monotherapy was related to improved OS in patients with PD-L1 in tumour infiltrating IC ≥ 5% (IC 2/3 on the VENTANA SP142 assay) plus high TMB (10 mut/Mb cut-off), when compared to placebo plus platin/gemcitabine, with an interim OS hazard ratio of 0.22 (95% CI, 0.08–0.63) [23].

Although PD-L1 and TMB are the most common biomarkers in many studies, some new strategies to predict response to ICI are being intensively investigated. Regarding the inflammatory status of the tumour, immune expression profiling is more accurate to determine a ‘hot’ tumour than PD-L1 expression alone. This technology, which quantifies gene expression from multiple cell types within a biopsy specimen, has shown a better correlation to chemokines, cytokines and cell surface proteins [35, 36]. For example, in the Checkmate-275 trial, there was a correlation between the presence of IFN-γ (Interferon gamma) gene signature and better response to Nivolumab in mUC (*p* = 0.0003) [53]. More recently, some Next-Generation RNA expression technologies allowed immune profiling of more than 700 genes [25].

Transcriptome profiling to classify bladder cancer in many distinct groups is also a promising technique to better predict responses. Some of these molecular subtypes of urothelial cancer were correlated to anti-PD-1/PD-L1 response in exploratory analyses [54–56]. For example, in IMvigor 210, the luminal cluster II subtype was correlated to a better response to Atezolizumab in mUC (ORR = 34%, *p* = 0.0017). This response was characterised by transcriptional signatures associated with presence of activated T-effector cells [26, 57]. On the other hand, Checkmate 275 showed discrepant results with better responses to Nivolumab in the Basal I subtype (ORR = 30%; luminal cluster II ORR = ~25%) [53]. These results show the difficulty to use the molecular subtypes as biomarkers, especially across agents, mostly because the molecular subtype criteria differ in each study and the best tissue to be biopsied (primary tumour or metastatic lesions) is not standardised [25].

Another growing biomarker is the evaluation of the mutational signature associated with the APOBEC family enzymes, present in approximately 80% of bladder cancers [58]. Despite having a different physiologic function correlated to anti-viral defence in normal cells, in the tumour cells these enzymes are likely correlated with hypermutation at cytosine bases in exposed single-stranded DNA [59]. NGS analyses, such as the TCGA and others, have identified that this mutational signature is characterised by a TCW > T/C mutation [58, 60, 61]. Tumours can be divided into APOBEC-high and low tumours. The first ones are more likely to have mutations in DNA damage response genes (TP53, ATR, BRCA2) and chromatin regulatory genes (ARID1A, MLL, MLL3), potentially leading to a hypermutation phenotype and subsequent enhanced immune response against the tumour [58]. For instance, in the IMvigor-130 trial, mUC patients with APOBEC mutational signature had significantly higher TMB and improved OS with atezolizumab containing regimens in the first-line cisplatin ineligible scenario [23]. Despite this promising correlation, more trials are still needed to confirm APOBEC high mutational signature as a reliable biomarker of response to immunotherapy, especially with other ICIs.

Table 1 summarises the main biomarkers in urothelial cancer and their features.

## Conclusion

As immunotherapy and other therapies arise as new opportunities for the treatment of MUC, there is an urgent need to identify predictive biomarkers in order to choose the best treatment for each patient. Lack of standardisation, difficulty to reproduce the tests and cost effectiveness are some of the challenges currently faced (Table 1). A comprehensive approach to analysis, including PD-L1, TMB and MSI, plus potential biomarkers of resistance is important to identify patient likelihood of response. Additionally, new and composite biomarkers are needed to better guide treatment with these new therapies.

## List of abbreviations

Anti-PD-1, Inhibitor programmed cell death protein-1; Anti-PD-L1, Inhibitor programmed death ligand-1; APOBEC, Apolipoprotein B mRNA editing catalytic polypeptide-like; CPS, Combined positive score; CTAs, Cancer-testis antigens; CR, Complete response; FDA, Food and Drug Administration; 18-F FDG PET/CT, Positron emission tomography/computer tomography with 2-deoxy-2[18-F]-fluoro-D-glucose; IC, immune cells; ICI, Immune checkpoint inhibitors; IHC, Immunohistochemistry; dMMR, Mismatch-repair deficient; MSI, Microsatellite instability; mUC, Metastatic urothelial cancer; Muts/Mb, Mutations per megabase; MVAC, Methotrexate, vinblastine, doxorubicin and cisplatin; NGS, Next-generation sequencing; NK cells, Natural killer cells; NSCLC, Non-small cell lung cancer; ORR, Overall response rate; OS, Overall survival; PD-1, Programmed cell death protein-1; PD-L1, Programmed death ligand-1; SUV max, Standardised uptake values maximum; TCGA, The Cancer Genome Atlas; TILs, Tumour-infiltrating lymphocytes; TMB, Tumour mutational burden.

## Clinical practice points

- **Immunotherapy has changed the paradigm of mUC treatment. There are five ICHs approved in first- and second-line of treatment**
- **We report a case of CR with pembrolizumab and a favourable molecular analysis that could be associated with this outstanding response**
- **To this day, there are not many reliable biomarkers that predict response to immunotherapy**
- **There is an urgent need to identify feasible and cost-effective predictive biomarkers of response to immunotherapy.**

## Conflicts of interest

Dr Bastos has received research funding from Janssen, Astellas and Pfizer, as well as honoraria and consulting fees from Janssen, Astellas, MSD, Bayer, Roche and AstraZeneca. Dr Lara Gongora has received speaker fees from MSD, Amgen, Janssen, Astellas, MSD, Bayer and AstraZeneca. The remaining authors declare that they have no known competing financial interests or personal relationships that could have appeared to influence the work reported in this paper.

## Ethical approval

Informed consent was obtained from the patient for publication of this case report and accompanying images.

## Authors' contributions

Marcello Moro Queiroz: writing original draft, online drafting, data collection.

Zenaide Silva de Souza: writing original draft, online drafting, data collection.

Aline Bobato Lara Gongora: writing-reviewing, supervision, methodology.

Felipe de Galiza Barbosa: writing-reviewing, figures development.

Carlos Alberto Buchpiguel: writing-reviewing, figures development.

Marilia Germanos de Castro: writing-reviewing, figures development, immunohistochemical analysis.

Mariana Petaccia de Macedo: writing-reviewing, figures development, immunohistochemical analysis.

Rafael Ferreira Coelho: writing-reviewing, methodology.

Ethan Samuel Sokol: writing-reviewing.

Anamaria Aranha Camargo: writing-reviewing, supervision.

Diogo Assed Bastos**:** conceptualization, writing-reviewing and supervision.

## Financial disclosure

This study was funded by Hospital Sírio-Libanês, São Paulo, Brazil.

## Figures and Tables

**Table 1.** Biomarkers in metastatic urothelial carcinoma and their main features.

Biomarkers	Method	Advantages	Disadvantages
PD-L1	Expression analysed by IHC	Rapid and low cost Widely applied in clinical trials	Distinct cut off values on each clinical study with different immunotherapies: varying from 1%, 5%, 10% and 25% Dynamic over time and space Does not represent the entire tumour microenvironment
PD-L1 (CD 274) amplification	NGS	Patients treated with ICI showed improved ORR and median progression-free survival	Need evaluation in the bladder cancer scenario Need prospective trials with a larger number of patients Did not always correlate with immunohistochemical High cost
TMB	Expression analysed by cancer-related genes panels (NGS)	Predict response to treatment based on immunotherapy in urothelial bladder cancer Related with the infiltration of T cells, NK cells and CTAs into the tumour microenvironment	Lack of standardisation regarding the definition of high TMB Some gene fusions, truncations and translocations may not be covered by NGS tests TMB can change depending on prior treatment Gene panel assays present differences in their methodology, the number of genes and types of mutations included
Immune expression profiling	Quantifies RNA from multiple cell types with next-generation RNA expression technologies	More accurate to determine ‘hot’ tumours Better correlation to chemokines, cytokines and cell surface proteins Next-generation RNA expression technologies with immune profiling greater than 700 genes	Needs results from phase III prospective studies there are still ongoing Multiple gene panels currently available High cost
Transcriptome profiling	Gene expression profiling based on TCGA Research Network	Correlation between responses to immunotherapy and some subtypes	Lack of standardisation, especially because molecular subtype criteria differ in each study Lack of standardisation in which is the best tissue to be biopsied (primary tumour or metastasis) Small cohorts until today Low negative predictive value (responses in all four subtypes) Does not assess the tumour microenvironment
APOBEC	NGS	Hypermutational phenotype and enhanced immune response APOBEC high correlated with better OS with some ICI	Benefit in OS with atezolizumab Need for more prospective trials with other ICI High cost

CTAs, Cancer-testis antigens; IC, Immune cell; IHC, Immunohistochemistry; NGS, Next-generation sequencing; TMB, Tumour mutational burden
